# Impact of COVID-19-Related Lockdown on Psychosocial, Cognitive, and Functional Well-Being in Adults With Down Syndrome

**DOI:** 10.3389/fpsyt.2020.578686

**Published:** 2020-10-28

**Authors:** Emanuele Rocco Villani, Davide Liborio Vetrano, Cecilia Damiano, Antonella Di Paola, Aurora Maria Ulgiati, Lynn Martin, John P. Hirdes, Laura Fratiglioni, Roberto Bernabei, Graziano Onder, Angelo Carfì

**Affiliations:** ^1^Centro Medicina Dell'Invecchiamento, Fondazione Policlinico Universitario Agostino Gemelli IRCCS and Università Cattolica del Sacro Cuore, Rome, Italy; ^2^Department of Neurobiology, Aging Research Center, Care Sciences and Society, Karolinska Institutet and Stockholm University, Stockholm, Sweden; ^3^Department of Neurology and Alzheimer Research Center, University of Groningen and University Medical Center Groningen, Groningen, Netherlands; ^4^Department of Health Sciences, Lakehead University, Thunder Bay, ON, Canada; ^5^School of Public Health and Health Systems, University of Waterloo, Waterloo, ON, Canada; ^6^Department of Cardiovascular, Endocrine-Metabolic Diseases and Aging, Istituto Superiore di Sanità, Rome, Italy

**Keywords:** COVID-19, lockdown, down syndrome, functioning, well-being

## Abstract

People with Down Syndrome (DS) have a high prevalence of physical and psychiatric comorbidities and experience early-onset dementia. With the outbreak of CoVID-19 pandemic, strict social isolation measures have been necessary to prevent the spreading of the disease. Effects of this lockdown period on behavior, mood and cognition in people with DS have not been assessed so far. In the present clinical study, we investigated the impact of CoVID-19-related lockdown on psychosocial, cognitive and functional well-being in a sample population of 46 adults with DS. The interRAI Intellectual Disability standardized assessment instrument, which includes measures of social withdrawal, functional impairment, aggressive behavior and depressive symptoms, was used to perform a three time-point evaluation (two pre-lockdown and one post-lockdown) in 37 subjects of the study sample, and a two time point evaluation (one pre- and one post-lockdown) in 9 subjects. Two mixed linear regression models – one before and one after the lockdown – have been fitted for each scale in order to investigate the change in the time-dependent variation of the scores. In the pre-lockdown period, significant worsening over time (i.e., per year) was found for the Depression Rating Scale score (β = 0.55; 95% CI 0.34; 0.76). In the post-lockdown period, a significant worsening in social withdrawal (β = 3.05, 95% CI 0.39; 5.70), instrumental activities of daily living (β = 1.13, 95% CI 0.08; 2.18) and depression rating (β = 1.65, 95% CI 0.33; 2.97) scales scores was observed, as was a significant improvement in aggressive behavior (β = −1.40, 95% CI −2.69; −0.10). Despite the undoubtful importance of the lockdown in order to reduce the spreading of the CoVID-19 pandemic, the related social isolation measures suggest an exacerbation of depressive symptoms and a worsening in functional status in a sample of adults with DS. At the opposite, aggressive behavior was reduced after the lockdown period. This finding could be related to the increase of negative and depressive symptoms in the study population. Studies with longer follow-up period are needed to assess persistence of these effects.

## Introduction

Down Syndrome (DS) is the most common genetic cause of developmental disability and cognitive impairment, with an incidence of about 1/800 live births (1). DS is also referred to as a “segmental” progeroid syndrome, with selected organ systems experiencing early aging and persons with this condition might present patterns of co-morbidities commonly observed in the older population (2). Moreover, people with DS start experiencing progressive cognitive impairment early in life, with a prevalence of dementia as high as 68–80% at the age of 65 years (3). The clinical picture of individuals with DS is often complicated by the presence of functional deficits, behavioral symptoms and nutritional and social problems, all of which have increased prevalence with age (4, 5). Sociality and social interactions are important for individuals with DS, who identify family involvement and affection as main supporting pillars in life (6). Interestingly, individuals with DS tend to have higher global scores for social adaptive skills compared to adults with other intellectual disabilities (ID) (7).

Despite the relatively high prevalence of DS in the general population, few data are available about the impact of Coronavirus disease-19 (COVID-19) among those with DS (8, 9). Concerns about the COVID-19 epidemic in this population are related to the presence of a dysfunctional immune system, possible exacerbations of psychiatric conditions and worsening of functional and cognitive impairment (10). With the pandemic outbreak, several countries including Italy implemented strict social isolation measures referred to as *lockdown*, to contain the contagion (11). The Italian government issued a provision to guarantee care to persons with disabilities during the lockdown period. In spite of that, a large part of the social assistance structures dedicated to people with DS and their caregivers had to drastically reduce their activities, depriving people with DS and their families/caregivers of effective support. This reduction of social, recreational and work activities during the lockdown may have impaired the physical and psychological resilience of the general population (12) and similarly may have triggered or exacerbated behavioral and mood changes or have worsened the global and cognitive functioning of adults with DS. However, no data are available yet on the effects of the lockdown in this vulnerable population. Therefore, the aim of the present study was to describe the impact of COVID-19-related lockdown on psychosocial, cognitive and functional well-being in a sample population of adults with Down syndrome.

## Methods

This clinical study included adults with DS, aged 18 years or older, followed at the outpatient clinic of the Geriatric Department of the Fondazione Policlinico Universitario A. Gemelli IRCCS, Università Cattolica del Sacro Cuore Rome, Italy since 2015 to date. Participants were referred to the clinic by DS associations and family physician and received a comprehensive medical assessment that included a multidimensional evaluation with the interRAI Intellectual Disability (InterRAI-ID) instrument (13–15).

The present study enrolled subjects with the following characteristics: adults with confirmed genetic diagnosis of DS, without severe intellectual disability (QI < 20) and either two InterRAI-ID evaluations from 2015 since the beginning of lockdown in Italy (11th of March 2020) or one InterRAI-ID evaluation within 6-months before the lockdown. Among these eligible participants, telephone-based interRAI-ID follow-up was performed after the lockdown (since 15th April 2020 to 31st May 2020) to all the persons/caregivers willing to participate.

The study was approved by the Ethical Committee of the Università Cattolica del Sacro Cuore. Informed consent was obtained from all participants. The surrogate legal representative was asked to get the information and give consent in those cases where individuals were unable to make the decision for themselves.

### Psychosocial, Cognitive, and Functional Assessment With the InterRAI-ID Instrument

Psychosocial, cognitive and functional well-being were evaluated through the interRAI-ID instrument, which contains over 350 data elements including socio-demographic variables, clinical items about physical and cognitive status, functioning, behaviors, and signs, symptoms, syndromes and treatments being provided (15). Items are compiled by a trained assessor based on history and basic signs and symptoms (e.g., face expressions, disruptive behaviors, pain frequency and intensity) collected directly from the individual being assessed, by an informant selected among the closest relatives (parents or siblings) or long-standing caregiver; a number of questions are asked directly to the individual concerning his or her preferences, outlook and well-being. Clusters of items are set up in algorithms and scales to deliver clinically relevant triggers to inform subsequent clinical evaluation. Such scales have proven internally consistent and valid among adults with ID (15). Cognitive status is evaluated through the Cognitive Performance Scale (CPS) (16) ranging from 0 (no cognitive impairment) to 6 (severe cognitive impairment). Functional status is evaluated through the 7-point Activities of Daily Living Hierarchy (ADLH), used to identify persons requiring assistance in ADLs (17), and through the 7-point Instrumental Activities of Daily Living Hierarchy (IADLH), used to identify those requiring assistance with IADLs (18). The two scales ranges from 0 (independent) to 6 (totally dependent). Depressive symptoms are assessed through the Depression Rating Scale (DRS), ranging from 0 to 14 with score ≥3 being indicative of depression DRS has been cross-validated with other scales such as the Hamilton Depression Scale (19). Aggressive behavior is assessed through the Aggressive Behavior scale (ABS), ranging from 0 to 12. A score from 1 to 4 defines mild/moderate aggressive behavior and scores ≥5 define severe aggressive behavior (20). The presence of negative symptoms, such as withdrawal from activities of interest, lack of motivation, reduction in social interaction or anhedonia, is evaluated through the Social Withdrawal Scale (SOCWD). Scores range from 0 to 12 with higher scores indicating higher levels of anhedonia (21). The presence of communication problems is evaluated through the Communication Scale (COMM), with score from 2 to 5 defining mild/moderate communication problems and scores from 6 to 8 defining severe communication problems (22). The PAIN scale scores pain in a 4-point scale, ranging from 0 (no pain) to 3 (severe daily pain) based on recollection by the person or the caregiver and is highly predictive of pain as measured by the Visual Analog Scale (23). ADLH and IADLH are coded according to the actual situation at the time of assessment while time frequencies of the items in the other scales are classified as present every day in the past 3 days, present in the past three days but not daily, present at least once in the last 30 days, not present (or present more than 30 days before the assessment).

### Analytical Approach

Sample characteristics were reported as mean and standard deviation (SD) or count and percentage (%). To compare the changes in the abovementioned scales before and after the lockdown, the follow-up time was centered around an index date, the day when the lockdown was established in Italy (11th March 2020). A value for each of the scales at the index date was predicted for each individual: assuming a linear change, for those with two available observations before the index date, we performed intra-subject linear regressions, considering time as predictor. Predicted values were rounded to the nearest integer. Since in the imputation procedure the variables were treated as continuous, the predicted values could fall outside the real range of variation. In this case, the predicted values have been approximated to the largest or smallest value belonging to the range, depending on the situation. For those individuals with only one observation preceding the lockdown, the predicted values at the index date were set as equal to the values observed previously (which were observed no more than 6 months before the lockdown). With the aim to evaluate the changes in participant's condition during the lockdown, a sign test for matched data was performed for all the considered scales. The test compares the distribution of the estimated values at the beginning of the lockdown with the distribution of the values observed afterwards. The null hypothesis was that the median of the estimated values at time 0 was equal to the median of the values detected during the lockdown period. Two mixed linear regression models – one before and one after the index date – have been fitted for each scale in order to investigate the change in the time-dependent variation of the variables. All models were adjusted by age and sex and a random effect was introduced at the intercept. A *p* < 0.05 was considered as statistically significant. Stata (StataCorp) 16.0 was used in all analyses.

## Results

Since 2015, a total of 221 adult individuals with DS were evaluated with the InterRAI-ID assessment in our clinic. We present data about 46 eligible individuals that agreed to participate to the telephone-based interRAI-ID follow-up after the lockdown. Nine of them had received an evaluation within 6 months before the lockdown and 37 had received two from 2015 until the lockdown. The characteristics of the study population before the lockdown are shown in Table 1. Mean age was 40.6 ± 13.3 years, 23 subjects were female (50%). Overall, 18 individuals (39.1%) were under the protection of a legal guardian and 9 (19.6%) were living with non-relative persons. On average they had received 43.0 ± 30.5 of informal care from family members, friends or neighbors in the 3 days before the evaluation. The most frequent medical conditions were visual impairment (87%), thyroid diseases (50%), hypoacusis (23.8%) and congenital cardiopathies (26.1%). Neuropsychiatric conditions were also prevalent: dementia was present in 8 persons (17.4%), 5 presented depression (10.9%), and 5 had language disorders (10.9%). One subject had autistic spectrum disorders. The mean number of regularly used drugs was 2.3 ± 2.0 and the mean number of psychotropic drugs was 0.5 ± 0.9.

**Table 1:** Sample characteristics before the lockdown.

	**Mean/count (SD/%)**
	***n* = 46**
Sex (female)	23 (50%)
Age (years)	40.6 (13.3)
**Residential status**	
Living at home	37 (80.4%)
Other (Institution, group home, etc.)	9 (19.6%)
Persons with legal guardian	18 (39.1%)
**Living arrangement**	
With parents or guardians	30 (65.2%)
With siblings	7 (15.2%)
With non-relatives	9 (19.6%)
Alcohol use (1 drink in last 14 days)	5 (10.9%)
BMI (Kg/m^2^)	26.0 (4.6)
**Medical conditions**	
Language disorders	5 (10.9%)
Cognitive decline	8 (17.4%)
Depression	5 (10.9%)
Autistic spectrum disorders	1 (2.2%)
Congenital cardiopathy	12 (26.1%)
Obesity	10 (21.7%)
Blood Cells abnormalities	7 (15.2%)
Visual impairment	40 (87%)
Hypoacusis	13 (28.3%)
Thyroid diseases	23 (50.0%)
Obstructive sleep apneas	7 (15.2%)
Osteoporosis	11 (23.9%)
Psoriasis	7 (15.2%)
Musculo-skeletal disorders	9 (19.6%)
N. of drugs	2.3 (2.0)
N. of psychotropic drugs	0.5 (0.9)
Informal care (hours in last 3 days)	43.0 (30.5)

Table 2 shows the mean scores of the investigated scales and the results of the sign test for the evaluation of changes in physical and mental health scales before and after the lockdown. The number of subjects that have worsened, improved or remained constant was significantly different for the IADLH scale (*p* = 0.003), for the ABS (*p* = 0.046), for the DRS (*p* = 0.032) and for the SOCWD scale (0.011).

**Table 2:** Mean scores of the scales before and after the lockdown and sign test for the evaluation of changes in participants condition during the lockdown.

	**Mean score of tests**^**a**^		**Sign test for changes**^**b**^			
	**Before**	**After**	**Worsening**	**Improvement**	**No**	***p*-value**
	**lockdown**	**lockdown**			**changes**	
ADLH	1.3 (1.5)	1.4 (1.3)	10	5	31	0.151
IADLH	3.9 (1.3)	4.2 (1.2)	11	1	34	**0.003**
ABS^c^	1.1 (1.4)	0.8 (1.0)	3	10	32	**0.046**
CPS	2.6 (0.8)	2.8 (1.0)	4	0	42	0.063
COMM	2.5 (1.4)	2.5 (1.3)	5	7	34	0.387
DRS	3.5 (2.0)	3.9 (1.7)	17	7	22	**0.032**
PAIN	0.5 (0.6)	0.4 (0.5)	0	3	43	0.125
SOCWD	0.8 (2.1)	1.6 (2.6)	13	3	30	**0.011**

a *Measures before lockdown refers to the values imputed at the beginning of the lockdown, while conditions after lockdown refers to the values observed afterwards*.

b *The test compares the estimated values at the start of the lockdown with those observed afterwards. For each scale, the number of subjects that have worsened, improved or remained constant is reported*.

c *There was no information regarding the value of the ABS variable after the lockdown for one of the study subjects. Therefore, that individual was not taken into consideration in the analysis of the ABS variable*.

*Bold values highlight parameters with statistically significant change*.

*SOCWD, Social withdrawal scale; ADLH, ADL hierarchy scale; IADLH, IADL hierarchy scale; COMM, Communication Scale; ABS, Aggressive Behavior scale; DRS, Depression Rating scale; CPS, Cognitive performance scale*.

**Figure 1:**
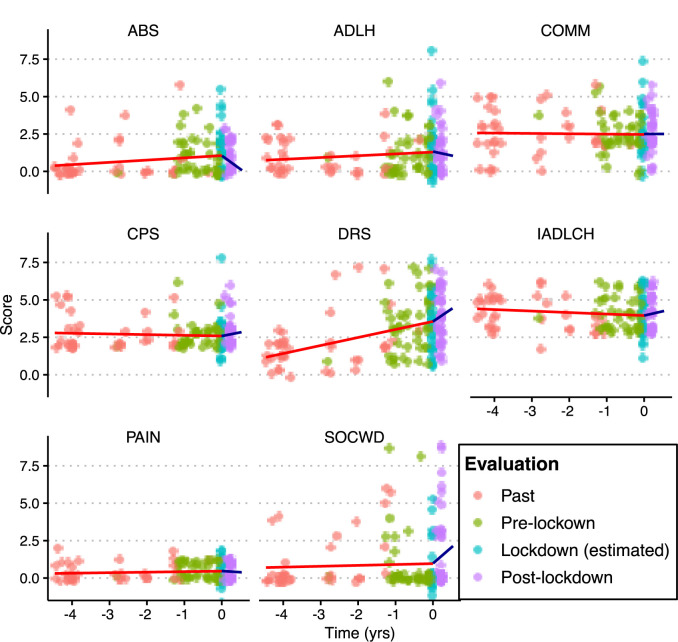
Rate of change over time of physical and mental health scales before and after the lockdown. Points represent the scores obtained by each individual in the different evaluation events. Fit line in the pre-lockdown phase in red color; fit line in the post-lockdown phase in blue color. The fit parameters are specified in Table 3.

Figure 1 and Table 3 shows the rate of change (β coefficient and 95% C.I.) over time of physical and mental health scales before and after the lockdown. Regarding the pre-lockdown period, a significant worsening over time (i.e., per year) was only found for the DRS score (β = 0.55; 95% CI 0.34; 0.76). Regarding the post-lockdown period, significant worsening in scores over time was found for the SOCWD scale (β = 3.05, 95% CI 0.39; 5.70), IADLH scale (β = 1.13, 95% CI 0.08; 2.18), and DRS (β = 1.65, 95% CI 0.33; 2.97), while a significant improvement was found for ABS (β = −1.40, 95% CI −2.69; −0.10). ADLH scale, CPS, COMM scale and PAIN scale did not show significant changes over time both during the pre-lockdown and in the post-lockdown period (*p* > 0.05 for all).

**Table 3:** Rate of change (β and 95% C.I.) over time of physical and mental health scales before and after the lockdown.

**Functional**	**Pre-lockdown**			**Post-lockdown**		
**scales**	**change per year**			**change per year**		
	**β**	**95% C.I**.	***p*-value**	**β**	**95% C.I**.	***p*-value**
SOCWD	0.04	−0.21; 0.30	0.742	**3.05**	**0.39; 5.70**	0.024
ADLH	0.09	−0.05; 0.25	0.208	0.23	−0.78; 1.24	0.651
IADLH	−0.10	−0.23; 0.02	0.112	**1.13**	**0.08; 2.18**	0.034
COMM	−0.03	−0.20; 0.14	0.759	−0.29	−1.23; 0.64	0.537
ABS	0.13	−0.03; 0.29	0.117	−**1.40**	−**2.69;** –**0.10**	0.034
DRS	**0.55**	**0.34; 0.76**	<0.001	**1.65**	**0.33; 2.97**	0.014
CPS	−0.03	−0.16; 0.09	0.595	0.55	−0.22; 1.31	0.164
PAIN	0.04	−0.03; 0.10	0.290	−0.26	−0.57; 0.05	0.106

*C.I., confidence interval*.

*Bold values highlight parameters with statistically significant change*.

*For all measures positive changes mean worsening and negative changes improvement in scale*.

## Discussion

The present study shows that social isolation measures related to COVID-19 lockdown reverberated on the functional and psychosocial well-being of adults with DS. To our knowledge, this is the first study describing the functional and psychosocial effects of the lockdown during the COVID-19 pandemic in people with DS.

Lockdown should be considered a potentially traumatic life-stressor event (24). Findings of our study should be discussed taking into account the adaptive behavior skills of individuals with DS. Throughout the lifespan, individuals with DS tend to demonstrate an adaptive behavior profile that involves relative strengths in receptive communication skills, domestic and community daily living skills and coping and interpersonal relationship socialization skills. Relative difficulties were reported in expressive and written communication (25). However, lower daily living coping skills and overall low adaptive behavior skills have been described for adults with DS when compared to age-matched general population individuals (26). Similar findings were found for elderly as compared to adult population {Cheng, 2014 #110}. In addition, in our sample, prevalence of visual and hearing impairment was high and sensorial deprivation can worsen adaptive behavior in adult individuals (27). But, it seems that vision problems do not decrease adaptive behavior skills in individuals with DS (28).

As expected from a lockdown-compliant population, our study sample showed a significant increase in social withdrawal scores (SOCWD) in the post-lockdown period. However, since the scale includes also dimensions other than social interactions indicators, the increase in SOCWD scores can also reflect an increase in anhedonia and lack of motivation. Notably, a high percentage of PTSD symptomatology, including anhedonia and sleep disturbances, was found also in a study including a sample of the general population in Italy (29). It is plausible that individuals with DS– frequently affected by neuropsychiatric conditions and dementia – may have been particularly prone to present such exacerbations.

We detected an increased depression burden during the post-lockdown period. Depressive symptoms are common among DS adults (30) and according to the pre-lockdown observation they appear to proceed faster than other measures. Yet, the time-dependent change in the DRS scores during the post-lockdown period was up to three time higher than pre-lockdown period, suggesting that stressor events (i.e., lockdown) could severely impact mood in individuals with DS (30). On the contrary, aggressive behavior scores (ABS) showed a significant decrease during post-lockdown period. A possible explanation of the decreased aggressive behavior observed in our study is that persons with DS are more likely to aggression toward peers or people who are not family members (31). Hence, social isolation could have reduced such external stimulation, resulting in a less demanding environment. On the other hand, it is known that catatonia and regression are frequent among young adults with DS facing stressful events (32), and internalized symptoms of depression emerge while externalized symptoms of aggressiveness decrease as they age (31). Indeed, social isolation in individuals with DS might have exacerbated or triggered negative symptoms (i.e., withdrawal, anhedonia, depression), while it could have mitigated aggressive behaviors.

From a functional point of view, there was a significant increase in IADL scores in the post-lockdown period, suggesting a decrease in independence in activities such as paying for things, shopping, and taking public transportation. On the one hand, this might be a consequence of the lockdown itself (compulsory stay-at-home policies, mandating closure of non-essential businesses), on the other hand it might have been the consequence of the disruption to their routines resulting in difficulties to understanding and adapting to the new requirements (such as wearing the face mask and respecting the contingent row at the supermarket), as has been described in the general elderly population (33). Conversely, the post-lockdown period did not show significant changes in ADL scores. This finding suggests that basic self-care activities such as dressing, washing and eating are less likely to be impaired by such stressor event.

The management of lockdown presents a perfect storm for mental distress for older people (34) and potentially even more for individuals with DS. Indeed, at any age individuals with ID present with significantly higher rates of mental health conditions when compared to the general population (35), and it is essential to thoroughly investigate their experience to devise effective ways of protecting them (10).

### Limitations

The present study has some important limitations. The study sample is small and with pre-lockdown evaluations spread out over a large timeframe. Furthermore, subjects in study were enrolled from an outpatient clinic, and could therefore be characterized by more complex health needs compared to the general DS population. As a consequence, the sample can't be considered to be representative of the population with DS and the results should be interpreted in the light of the small sample size and the possible selection bias. Finally, although InterRAI-ID is validated both for in person and on the phone administration, the different routes of administration pre- and post-lockdown could have introduced further bias.

## Conclusion

Despite the undoubtful importance of the lockdown in order to reduce the spreading of the COVID-19 pandemic, the related social isolation measures seemed to exacerbate depressive symptoms and some functional impairment in a population of adults with DS. Instead, aggressive behavior was less incident and could be related to the increase of negative and depressive symptoms. In light of such evidence, it will be important to assess in future studies the possible presence of long-term effects on the health of individuals with DS and how the disruptions of their routine affected not only other individuals with ID but also their caregivers. Doing this could lead to more awareness and to a novel insights in possible assistance and treatment strategies.

## Data Availability Statement

The raw data supporting the conclusions of this article will be made available by the authors, without undue reservation.

## Ethics Statement

The studies involving human participants were reviewed and approved by Ethical Committee of Università Cattolica del Sacro Cuore. The patients/participants provided their written informed consent to participate in this study.

## Author Contributions

GO and RB designed and supervised the study. AP, EV, and AC assessed the participants and managed the dataset. DV and CD conceived and carried out the data analysis. EV and AC drafted the manuscript and coordinated the writing. GO, DV, AU, RB, LM, LF, and JH critically revised the manuscript. All authors contributed to the article and approved the submitted version.

## Conflict of Interest

The authors declare that the research was conducted in the absence of any commercial or financial relationships that could be construed as a potential conflict of interest.
